# Restorative Effects of Inulin From *Codonopsis pilosula* on Intestinal Mucosal Immunity, Anti-Inflammatory Activity and Gut Microbiota of Immunosuppressed Mice

**DOI:** 10.3389/fphar.2022.786141

**Published:** 2022-02-14

**Authors:** Yuan-Feng Zou, Cen-Yu Li, Yu-Ping Fu, Xin Feng, Xi Peng, Bin Feng, Li-Xia Li, Ren-Yong Jia, Chao Huang, Xu Song, Cheng Lv, Gang Ye, Ling Zhao, Yang-Ping Li, Xing-Hong Zhao, Li-Zi Yin, Zhong-Qiong Yin

**Affiliations:** ^1^ Natural Medicine Research Center, College of Veterinary Medicine, Sichuan Agricultural University, Chengdu, China; ^2^ Animal Nutrition Institute, Sichuan Agricultural University, Chengdu, China; ^3^ Key Laboratory of Animal Disease and Human Health of Sichuan Province, College of Veterinary Medicine, Sichuan Agricultural University, Chengdu, China; ^4^ Institute of Ecological Agriculture, Sichuan Agricultural University, Chengdu, China

**Keywords:** inulin-type fructan, *Codonopsis pilosula*, immune-enhancement, antiinflammatory, gut microbiota

## Abstract

An inulin (CPPF), isolated from a traditional Chinese herbal medicine *Codonopsis pilosula*, was characterized and demonstrated with potential prebiotic activity *in vitro* before. Based on its non-digested feature, the intestinal mucosa and microbiota modulatory effects *in vivo* on immunosuppressed mice were investigated after oral administration of 200, 100 and 50 mg/kg of CPPF for 7 days. It was demonstrated that the secretions of sIgA and mucin 2 (Muc2) in ileum were improved by CPPF, and the anti-inflammatory activities in different intestine parts were revealed. The intestine before colon could be the target active position of CPPF. As a potential prebiotic substance, a gut microbiota restorative effect was also presented by mainly modulating the relative abundance of *Eubacteriales*, including *Oscillibacter*, *unidentified Ruminococcus* and Lachnospiraceae after high-throughput pyrosequencing of V4 region of 16S rRNA analysis. All these results indicated that this main bioactive ingredient inulin from *C. pilosula* was a medicinal prebiotic with enhancing mucosal immune, anti-inflammatory and microbiota modulatory activities.

## Introduction

The inulin, consisting of fructose units linked by β-(2→1) bonds and a terminal glucose by α-(1→2) bond, remains relatively intact after transition through the digestive system (Shoaib et al., 2016). It was demonstrated with effects of improving immune and promoting the proliferation of beneficial bacteria, which are related with their degree of polymerization and plants species (Moreno-Vilet et al., 2014; García Gamboa et al., 2018). All these benefits are related to body health through positively balancing gut microbiota via increasing the abundance of beneficial bacteria and reducing potential pathogens. The pathogens colonization were inhibited by the antagonism from organic acids or inhibitory peptides (García Gamboa et al., 2018). Inulin is also identified with anti-obesity, anti-diabetes, anti-inflammatory, anti-hypertension, anti-oxidative and anti-cancer activities, as well as exerts abilities to control inflammatory bowel disease, promote colonic absorption of minerals and stimulate the immune system (Karimi et al., 2015; Vogt et al., 2015; Ahmed and Rashid, 2017; Shang HM et al., 2018; Mazraeh et al., 2019). These activities support inulin a widely use as function foods on reducing cardiovascular disease, lowering blood urea and uric-acid levels (Karimi et al., 2015).

The roots of *Condonopsis pilosula* (Radix Codonopsis) has been used in Traditional Chinese Medicine (TCM) for hundreds of years. Three species of its plant sources are recorded in the Pharmacopoeia of the People’s Republic of China, including *Codonopsis pilosula* (Franch.) Nannf., *C. pilosula* Nannf. var. *modesta* (Nannf.) L. T. Shen and *C. tangshen* Oliv. (Chinese Pharmacopoeia Committee, 2020), for the treatment of nourishing the spleen and stomach and tonifying *Qi* of stomach. Modern pharmacological studies have uncovered Radix Codonopsis with effects like immunomodulation, anti-ulcer, lowering blood pressure, as well as intestinal flora modulation. It has also been widely used as nutritional supplement in China on homology as both medicine and food (Gao et al., 2018). The polysaccharide fraction is considered as a potential bioactive natural product possessing modulatory effect on immunity and intestinal microbiota (Fu et al., 2018a; Peng et al., 2018; Deng et al., 2019; Zou et al., 2019). Similar functions of polysaccharides from *C. pilosula* Nannf. var. *modesta* (Nannf.) L. T. Shen (CPP), have been reported in our previous studies, like decreasing the levels of interferon-γ (IFN-γ), interleukin-2 (IL-2), IL-10, elevating serum ileum IgG, intestinal secretory immunoglobulin A (sIgA), and the amount of *Lactobacillus* and acetic acid content in cecum on cyclophosphamide (CY)-induced immunosuppressed mice (Fu et al., 2018a). And inulin, a reserve carbohydrate of Radix *Codonopsis,* has also been identified with high proportion (62%, w/w) as the neutral composition of CPP, with degree of polymerization (DP) of 2–17, and exerted underlying prebiotic activity *in vitro* (Fu et al., 2018b). Therefore, we deduced that the inulin as the main bioactive component of CPP, acts on intestinal mucosal improvement and microbiota regulation. The active target organ is possibly the intestine due to its non-digested property and the conclusions in our experimental work mentioned above. Moreover, the fermentable properties of inulin are related to their chemical structure, especially Mw (Gibson and Delzenne, 2008). There is a possibility that certain short inulin could have been digested completely when it arrives specific intestinal positions, which is seldom proofed by study of inulin from natural herbs. It is consequently of interest to figure out its efficacy on anti-inflammation in different intestinal segments, which could be helpful for the clinical application of inulin on various intestinal inflammatory disease.

Therefore, in this study, the intestinal modulatory effects of inulin from *C. pilosula* was evaluated from aspects of intestinal mucosal immunity, anti-inflammatory and gut microbiota modulatory functions on immunosuppressed mice. Among them, a comparison of anti-inflammatory with different intestinal segments was investigated in order to find the target intestine location.

## Materials and Methods

### Materials and Chemicals

The roots of *C. pilosula* Nannf. var. *modesta* L. T. Shen were collected in October 2017 from Jiuzhaigou County (Tibetan Qiang Autonomous Prefecture of Ngawa, China), and identified by Yuan-Feng Zou, College of Veterinary Medicine, Sichuan Agricultural University. The roots were dried and pulverized to a fine powder, and the fructan was obtained by DEAE-sepharose gel chromatograph, and identified as inulin-type fructan (the fructan form *C. pilosula*, CPPF) (Fu et al., 2018b).

The cyclophosphamide (CY, C8650) was obtained from Solarbio technology Co., Ltd., (Beijing, China). The ELISA kits, including mouse IL-1β, tumor necrosis factor-α (TNF-α), sIgA and mucin 2 (Muc2), were purchased from Enzyme-linked Biotechnology Co., Ltd., (Shanghai, China). The acetic acid (71251), propionic acid (94425) and butyric acid (19215) standards were purchased from Sigma-Aldrich (St. Louis, MO, United States). The primeScript RT reagent kit (with gDNA Eraser, RR047A), the TB Green Premix Ex Taq II (Tli RNaseH Plus, RR821A) and Trizol RNA isolation reagent were obtained from TAKARA, Japan. All other chemicals, such as chloroform, isopropanol, etc., were of analytical grade, obtained from the Chengdu Kelong chemical factory (Chengdu, China).

### Animal Care and Experimental Design

Sixty male specific-pathogen-free C57BL/6 mice (6–8 weeks old) were purchased from Beijing Vital River Laboratory Animal Technology Co., Ltd (Beijing China). They were maintained in a specific pathogen-free environment, where the temperature was 25 ± 2°C, with humidity of 60%. All mice were acclimatized for 7 days with an automatically-controlled 12 h light/dark cycle and free access to sterile food and distilled water.

Although CY is an alkylating drug used on cancer and autoimmune disease, it has been used as an immunosuppression-inducing agent due to its major side effect (Wang Z et al., 2019). In this study, a stable and classical CY-induced immunosuppression model was used to evaluate the recovery effect of CPPF on both intestinal immunity and microbiota modulation, according to several previous studies (Tang et al., 2018; Zhu et al., 2018; Wang Z et al., 2019). The mice were divided into five groups, 12 mice each. The immunosuppressed mice (4 groups) were given 60 mg/kg (0.1 ml/10 g body weight) CY for 3 days *via* intraperitoneal injection, once a day (Fu et al., 2018a). The normal mice were injected with 0.1 ml/10 g body weight saline as Control group. After 3 days of CY/saline treatment, the mice were administrated orally with freshly prepared CPPF (dissolved in saline) at dosage of 200, 100, 50 mg/kg (dosages were set in line with that of CPP in our previous study (Fu et al., 2018a) or saline (0.1 ml/10 g body weight) for 7 days in succession, as CY + CPPF-H, CY + CPPF-M, CY + CPPF-L and Control/CY groups, respectively. After 24 h of the last administration, all mice were euthanized with carbon dioxide followed by cervical dislocation, and the different segments of intestinal tissues, and the content of cecum were separated immediately and stored at −80°C.

### Determination of the Intestinal Cytokines, sIgA and Muc 2

IL-1β and TNF-α secretions in intestine tissues (including jejunum, ileum, and colon), and sIgA, Muc2 in ileum, were determined using ELISA kits. The intestinal tissues were ground in liquid nitrogen and homogenized in saline (50 mg/ml). The supernatants after centrifugation at 2,862 g for 20 min, 4°C, were collected and determined according to the manufacturer’s instructions.

### Quantitative Real-Time PCR

RNA extraction from jejunum, ileum and colon tissues (without content) and the quantitative real-time PCR (qRT-PCR) were performed as previously reported (Huang et al., 2014). Briefly, total RNA was extracted using Trizol reagent, and the quality and nucleic acid concentration were measured using spectrophotometer (NanoDrop 2000; Thermo Scientific, Shanghai, China). Reverse transcription was processed according to the manufacturer’s instructions (two-step). Quantitative real-time PCR was performed using the Bio-Rad CFX-96 system and TB Green Premix Ex Taq II kit. Gene expressions were normalized related to β-Actin, with primer sequences for SYBR Green probes of NGB obtained from Primer Bank: β-actin (No. 6671509a1), IL-1β (No.118130747c1) and TNF-α (No.133892368c2).

### Bacteriological Analysis and the Determination of Short-Chain Fatty Acids

The intestinal digesta of cecum were isolated after last gavage, and divided into two parts stored at −20°C (for SCFAs determination) and −80°C (for the bacteriological analysis). The samples stored at −80°C were transported to Beijing Novogene Science and Technology Co., Ltd. under dry-ice cooling environment. The DNA was extracted using CTAB/SDS method, and diluted to 1 ng/μL using sterile water. The V4 region of 16S rRNA was amplified through PCR using specific primer (515F: CCTAYGGGRBGCASCAG; 806R: GGACTACNNGGGTATCTAAT) with the barcode, then purified with GeneJETTM Gel Extraction Kit (Thermo Scientific). Sequencing libraries were generated using Ion Plus Fragment Library Kit 48 rxns (Thermo Scientific) following manufacturer’s recommendations. The library quality was assessed on the Qubit@ 2.0 Fluorometer (Thermo Scientific). The library was sequenced on an Ion S5^TM^ XL platform and 400 bp/600 bp single-end reads were generated. Sequences analysis were performed by Uparse software (Uparse v7.0.1001), and the analysis of diversity were calculated with QIIME (Version1.7.0) and displayed with R software (Version 2.15.3). The concentrations of SCFAs, including acetic acid, propionic acid and butyric acid, were detected on gas chromatography (GC) after several procedures for pretreatment based on the method of previous study (Fu et al., 2018a). They were quantitated according to the standard curve with crotonic acid as internal standard.

### Statistical Analysis

The SPSS 22.0 software was used to carry out a One-way ANOVA test, followed by LSD post-hoc test. All the experimental data were expressed as Mean ± SD (‾x ± SD) in table and Mean ± SEM (‾x ± SEM) in figures. The *p*-value of 0.05 or less was considered with statistically significance.

## Results

### The sIgA and Muc2 Secretion Were Promoted by CPPF

**FIGURE 1:**
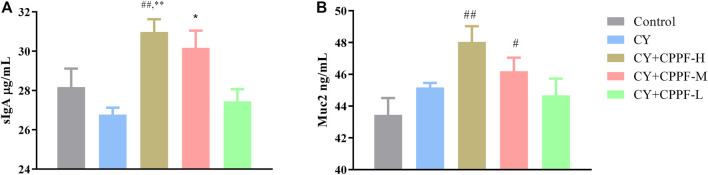
The effect of CPPF on sIgA (**A**) and Muc2 (**B**) secretion in ileum tissue; * indicated significant difference compared with CY group, *p* < 0.05; ** indicated significant difference compared with CY group, *p* < 0.01^; #^ indicated significant difference compared with Control group, *p* < 0.05; ^##^ indicated significant difference compared with Control group, *p* < 0.01; *n* = 12.

As the basic and essential immune antibody secreted in intestinal mucosa, sIgA could be promoted by polysaccharides from plenty of traditional medicines (Huang X et al., 2017; Sheng et al., 2017). It was demonstrated that CPPF, in both CPPF-H and CPPF-M groups, significantly promoted the secretion of sIgA in ileum (*p* > 0.05), even though it was not inhibited by CY, as shown in Figure 1A. Mucin, one of the chemical components in intestinal immune system, acts as the main line of defense to protect the epithelial cells from plenty of microbiota in the gut lumen (Kim and Ho, 2010). It was also revealed that the Muc2 secretion in ileum was not influenced by CY, but was facilitated markedly by CPPF in a dosage-dependent manner, as shown in Figure 1B. These results suggested that CPPF could play a promotive effect on intestinal mucosal immune system.

### The Anti-Inflammatory Effect of CPPF in Intestine

CY has been demonstrated with an induction of inflammatory reaction, as well as damages on intestinal mucosa and microbiota balance (Shang et al., 2016; Lee and Kang, 2017). While, the biological functions of inulin have been reported through anti-inflammation and modulation on gut microbiota as prebiotics (Li et al., 2019). However, the degradation status of inulin in intestinal tract is unknown so far, and whether its biological activities would be affected is also unclear. We investigated a polysaccharide from *Ligusticum chuanxiong*, the LCP-II-I, that it only displayed antioxidant activity well before colon *in vivo* (Huang C et al., 2017). Thus, the anti-inflammatory effect of CPPF on different intestinal segments were assessed, including jejunum, ileum and colon, in both mRNA and protein expression aspects.

**FIGURE 2:**
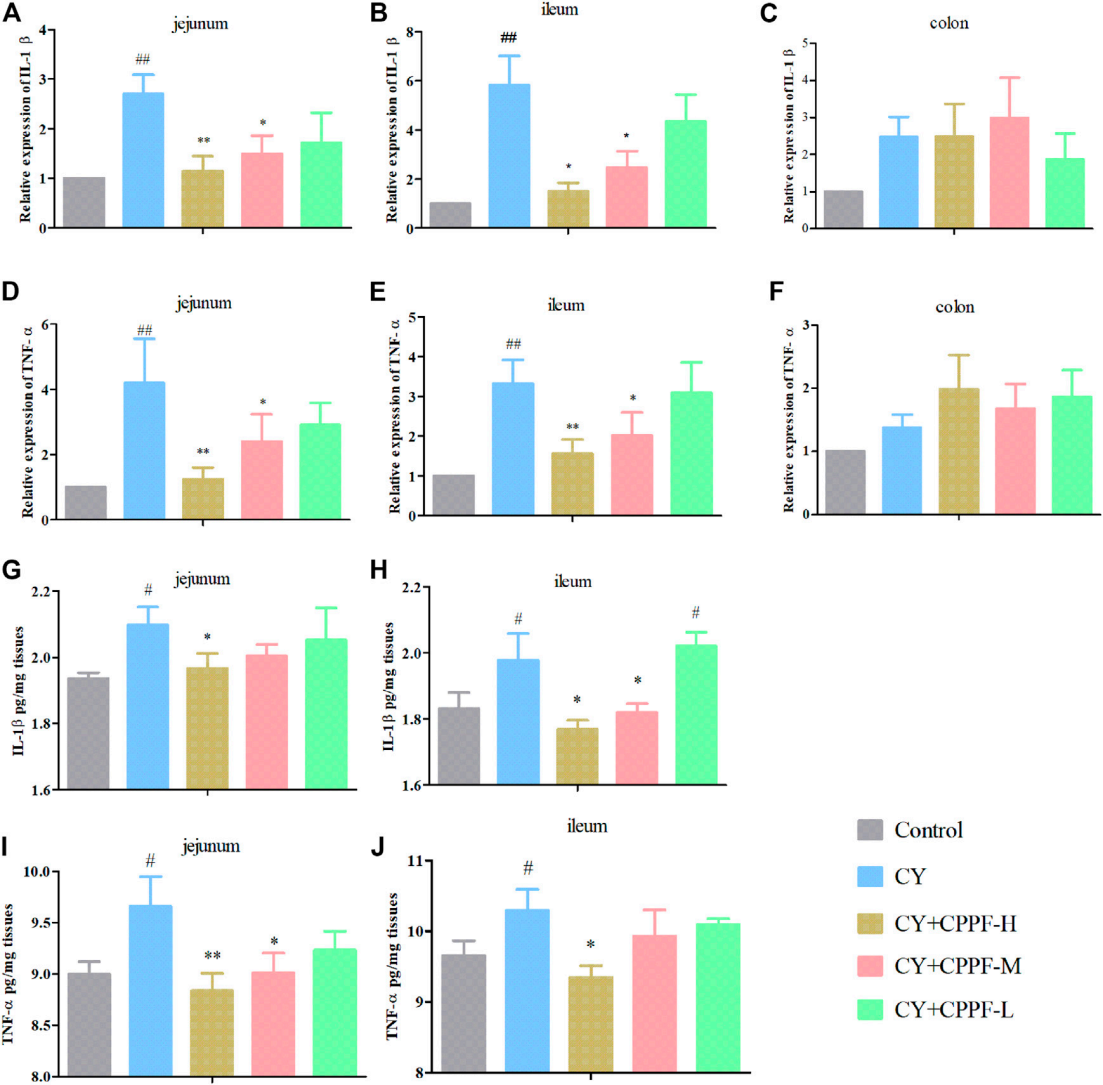
The effect of CPPF on IL-1β (**A–C,G,H**) and TNF-α (**D–F,I,J**) mRNA and protein expression in different parts of intestine tissue; * indicated significant difference compared with CY group, *p <* 0.05; ** indicated significant difference compared with CY group, *p <* 0.01; ## indicated significant difference compared with Control group, *p <* 0.01; # indicated significant difference compared with Control group, *p <* 0.05, *n* = 12.

The gene expressions of IL-1β and TNF-α were significantly increased both in jejunum and ileum after CY injection (*p* < 0.05) compared with the Control group (Figures 2A,B,D,E), except colon (*p* > 0.05) (Figures 2C,F). However, CPPF at high and medium dosage inhibited these inflammatory reactions induced by CY (*p* < 0.05), especially at high dosage group (*p* < 0.01). But, these suppression didn’t appear in colon, as shown in Figure 2. Given a poor expression and lower recovery effect by CPPF in colon, the protein secretion of IL-1β and TNF-α were not determined in further studies. Being consistent with their mRNA expression, the protein secretions of IL-1β and TNF-α in jejunum and ileum were enhanced remarkably after CY treatment, and were restored by CPPF (Figures 2G–J), especially that in CPPF-H group. Hence, it was revealed that the CPPF exerted an anti-inflammatory effect on immunosuppressed mice, and only worked before colon.

### The CPPF Modulated the Gut Microbiota Composition

#### The Operational Taxonomic Units, α-Diversity and β-Diversity Analysis

**FIGURE 3:**
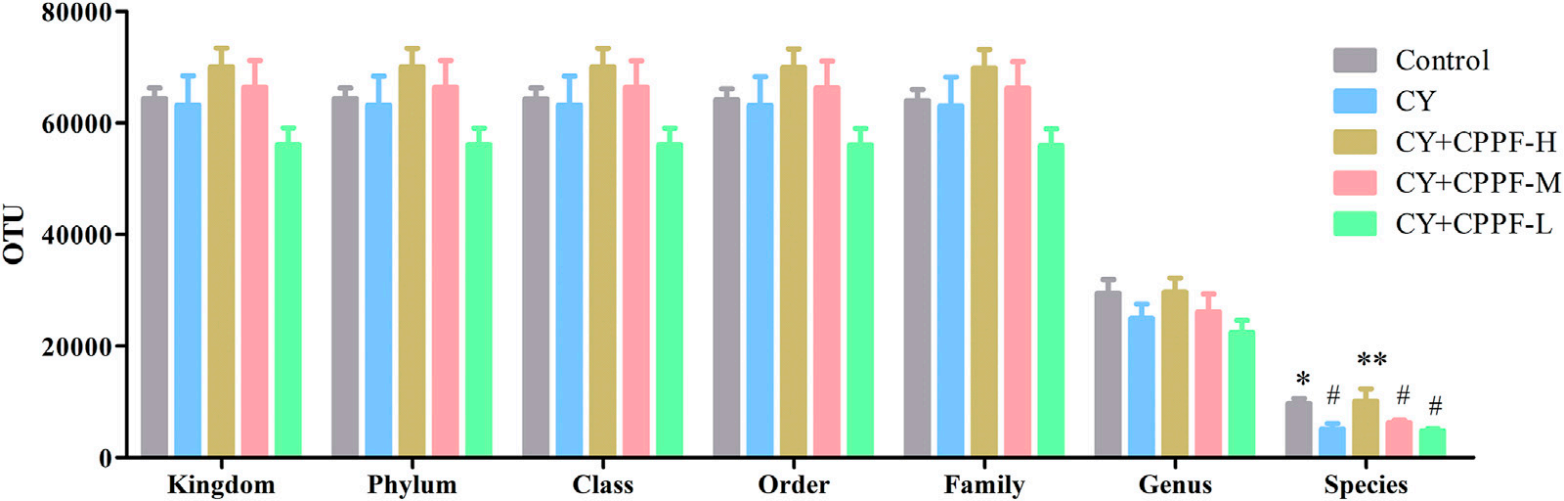
The Operational Taxonomic Units (OTUs) at Kingdom, phylum, class, order, family, genus and species levels of cecal content. * indicated significant difference compared with CY group, *p <* 0.05; ** indicated significant difference compared with CY group, *p <* 0.01; # indicated significant difference compared with Control group, *p <* 0.05; *n* = 7.

In order to study the bacterial composition of immunosuppressed mice, the OTUs (97% consistency) were clustered on the effective tags of all samples, and species annotation was further performed on the representative sequences of OTUs, as shown in Figure 3. It was classified and counted after removing low-quality sequences, and indicated that CPPF showed a trend of increasing OTUs at different levels compared with CY group (*p* > 0.05). Among them, OTUs value was reduced by around 50% in CY group at species level compared with that in Control group (*p* < 0.05), and was recovered after 200 mg/kg CPPF administration (*p* < 0.05). The other two CPPF groups also showed a recovery trend in a certain extent (*p* > 0.05).

**FIGURE 4:**
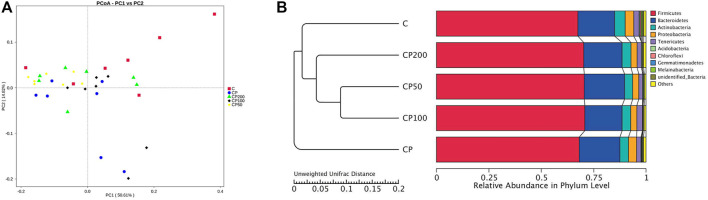
PCoA **(A)** and UPGMA **(B)** analysis; C was the Control group, CP was the CY group, CP200 was the CY + CPPF-H group, CP100 was the CY + CPPF-M group, and CP50 was the CY + CPPF-L group; *n* = 7.

All the differences and similarities of bacterial communities between groups were revealed by the weighed UniFrac distances at the phylum level, and displayed by PCoA analysis (Figure 4A). Based on the reduced observed species, ACE and PD whole tree index in α-diversity (Table 1), CY was suggested with an obvious regulation relatively to control group (*p* < 0.05), and a recovery trend by CPPF was also observed (*p* > 0.05). The Unweighted Pair-Group Method with Arithmetic Mean (UPGMA) was used to study the similarities and cluster analysis, which was showed as a systematic clustering tree (Figure 4B). It was exhibited that the bacteria belonging to *Firmicutes* were promoted after CPPF supplement. While, the bacteria belonging to *Bacteroidetes* were regulated in any group (*p*>0.05). The relative abundance of other phylum, like *Acidobacteria*, *Chloroflexi*, *Gemmatimonadetes*, *Melainabacteria* in CY treated groups, differed with those in control group, which also demonstrated a microbial dysbiosis affected by CY.

**TABLE 1:** The statistical results of α-diversity indices.

	Control	CY	CY + CPPF-H	CY + CPPF-M	CY + CPPF-L
observed_species	498.43 ± 52.43	458.86 ± 49.54^a^	464.86 ± 19.66	462.57 ± 16.70	463.71 ± 29.10
ACE	536.20 ± 51.91	494.55 ± 50.42^a^	503.95 ± 24.95	500.60 ± 24.49	496.49 ± 33.57
PD_whole_tree	36.25 ± 4.17	31.54 ± 3.91^a^	32.42 ± 3.40	33.41 ± 4.48	31.02 ± 4.00^a^

*p <* 0.05; it was indicated that there was dramatically decrease of alpha diversity in CY, group compared with control group.

a Indicated significant difference compared with Control group.

#### The Modulation of Gut Microbiota Composition by CPPF

**FIGURE 5:**
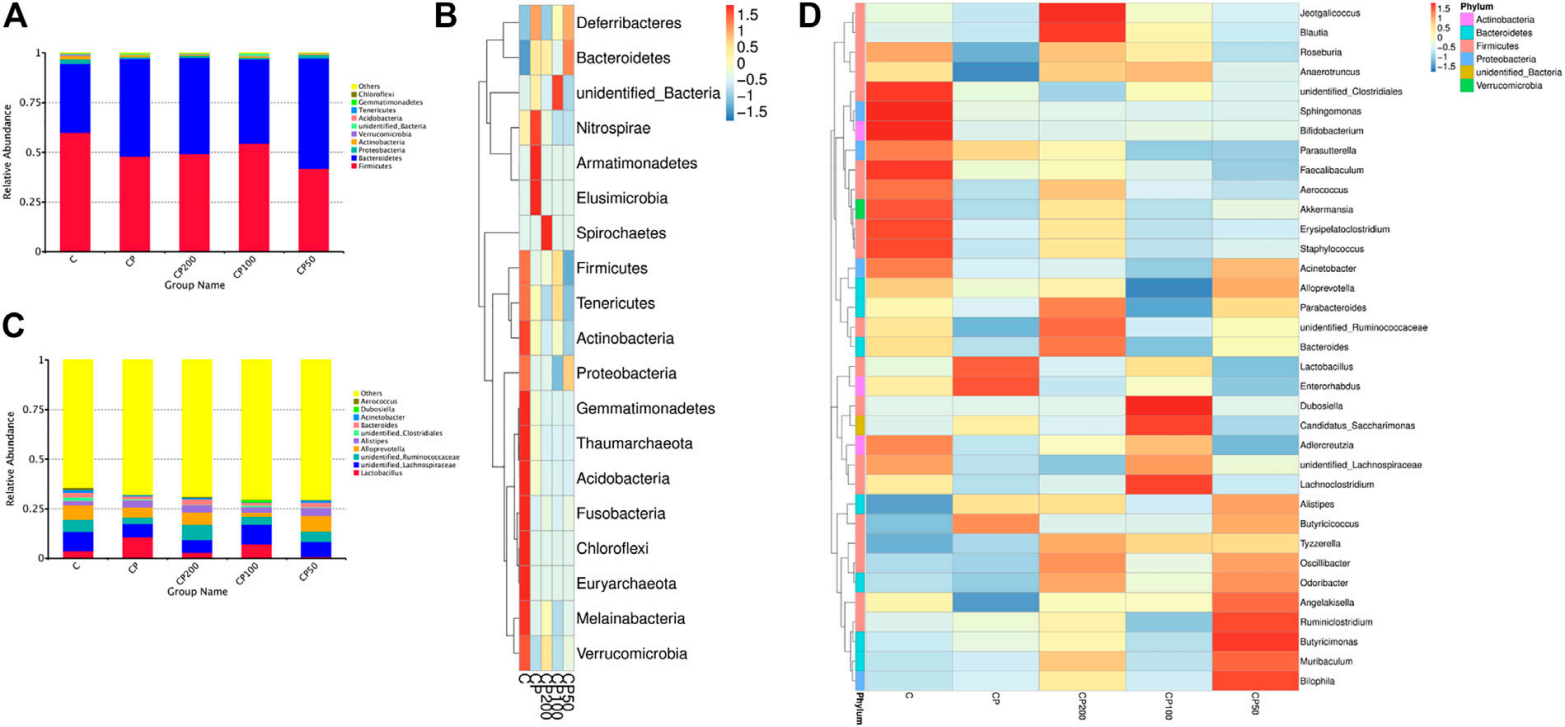
The histograms and heatmaps of bacterial composition change at phylum **(A,B)** and genus level **(C,D)**; C was the Control group, CP was the CY group, CP200 was the CY + CPPF-H group, CP100 was the CY + CPPF-M group, and CP50 was the CY + CPPF-L group; *n* = 7.

**FIGURE 6:**
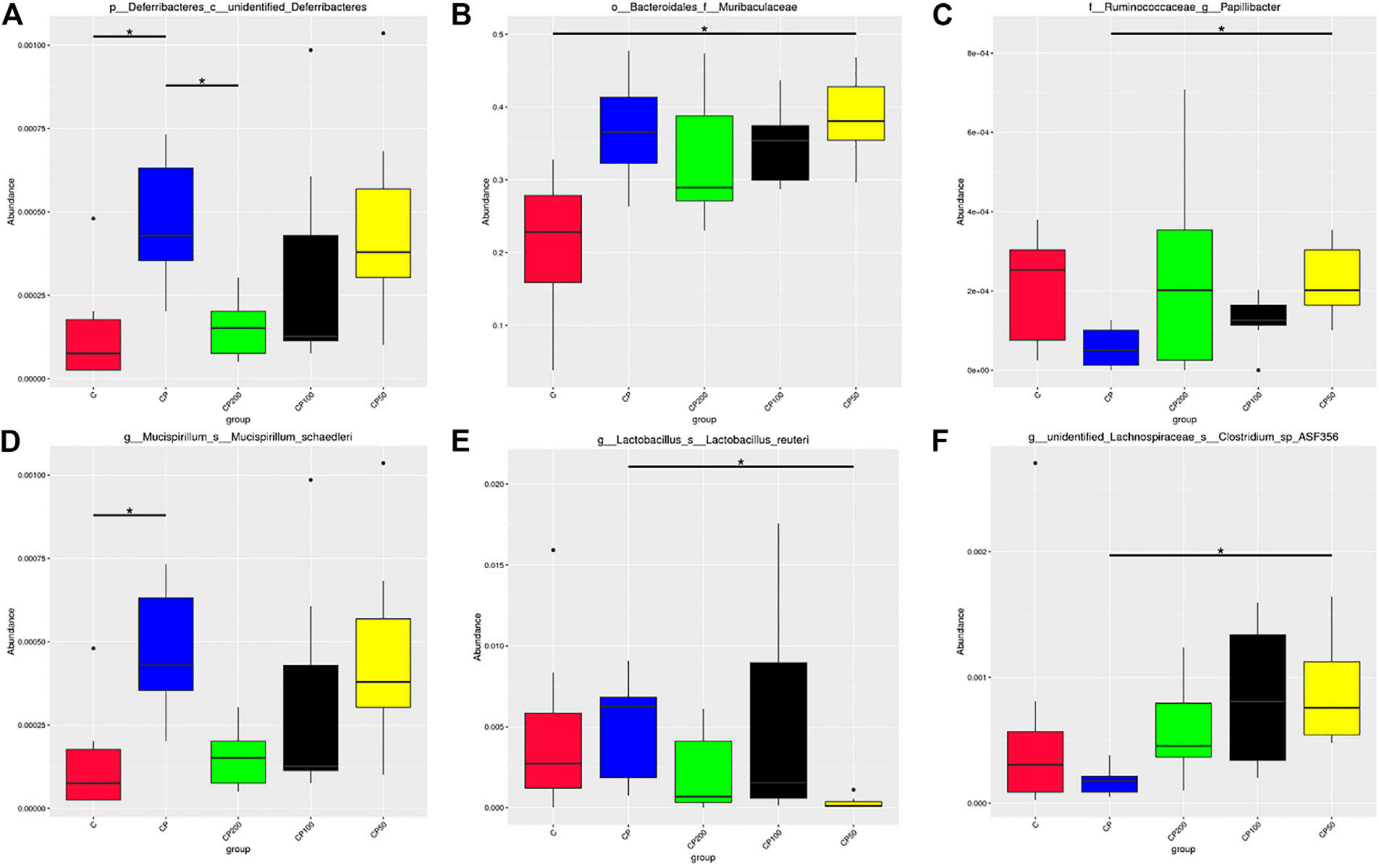
Species with significant differences at the level of class **(A)**, family **(B)**, genus **(C)**, and species **(D–F)**; * indicated that the difference between groups was significant *p <* 0.05; C was the Control group, CP was the CY group, CP200 was the CY + CPPF-H group, CP100 was the CY + CPPF-M group, and CP50 was the CY + CPPF-L group; *n* = 7.

The relative abundance of top 10 bacteria was calculated based on the species annotation and classification, and expressed as histograms and heatmap both at phylum and genus levels in Figure 5. The main bacteria compositions of immunosuppressed mice were changed, showing a remarkable lower relative abundance of *Firmicutes* (like *Erysipelatoclostridium, Lachnoclostridium, Unidentified* Ruminococcaceae*,* and Ruminococcaceae*,* Supplementary Figures S1, S2), *Melainabacteria*, and *Actinobacteria* (*p >* 0.05), and a higher abundance of *Bacteroidetes* (like family Muribaculaceae*,* Supplementary Figure S2) and *Deferribacteres* (*p <* 0.05, Figure 6A). While the bacteria of *Firmicutes* phyla, such as genus *Unidentified* Ruminococcaceae, *Oscillibacter* (Supplementary Figures S3, S4), *Lachnoclostridium, Anaerotruncus,* and *Angelakisella* (Supplementary Figures S4, S5) were enhanced dramatically in mice treated with CPPF (*p <* 0.05), and those of *Bacteroidetes* were notably reduced, such as genus *Alloprevotella* (Supplementary Figure S5, *p <* 0.05).

The difference of microbial community structure was analyzed by the analysis of similarities (Anosim), MRPP and AMOVA (data not shown), the species with significant change on abundance at different levels among those five groups were found by MetaStat method, as presented in Figure 6. It was demonstrated that CY has led to a gut microbiota imbalance, primarily altered the bacterial abundance of *Deferribacteres* (Figure 6A), Muribaculaceae (Figure 6B), *Papillibacter* (Figure 6C), *Lactobacillus reuteri* (Figure 6D), *Mucispirillum schaedleri* (Figure 6E), and *Clostridium* sp ASF356 (Figure 6F). However, after supplementation of CPPF, the abundance of bacteria of *Deferribacteres*, *Papillibacter* and *Clostridium* sp ASF356, were recovered prominently within just 7 days of treatment, compared with CY or Control group (*p <* 0.05), which indicated a promising modulatory effect of CPPF on gut bacterial community.

**FIGURE 7:**
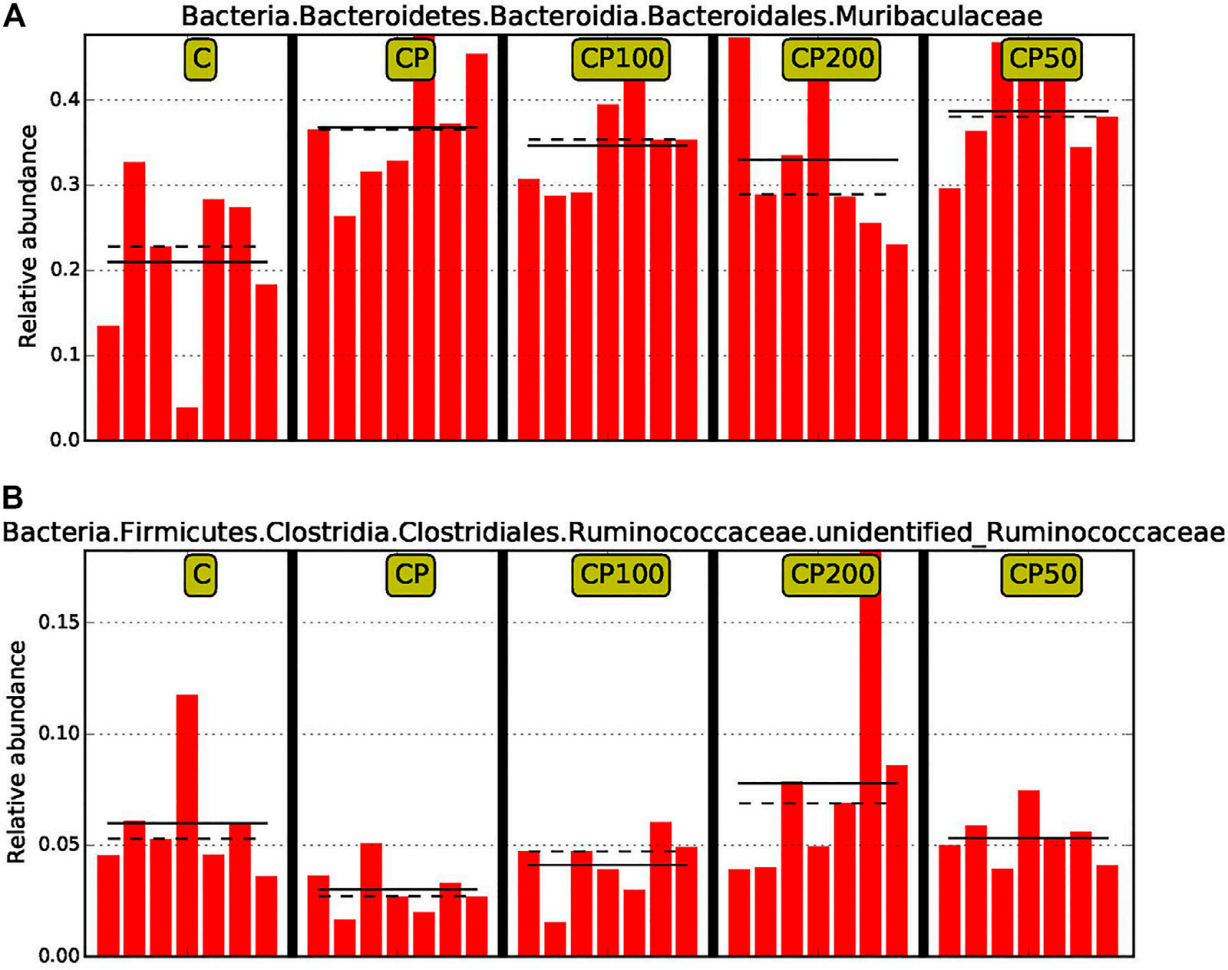
The comparison of relative abundance of biomarkers with statistical differences between groups based on the LefSe results. The highest abundance of each biomarker in the sample was set as 1, while the rest samples were expressed by the value of relative abundance to the highest abundance sample. The solid line and dotted line represented the mean and median of the relative abundance of each sample respectively; C was the Control group, CP was the CY group, CP200 was the CY + CPPF-H group, CP100 was the CY + CPPF-M group, Q18 and CP50 was the CY + CPPF-L group; n = 7.

Moreover, in order to discover and interpret the high-dimensional biomarkers, the LEfSe (LDA Effect Size) was used to find the statistical differences among groups. The significantly different species were picked out after analysis with LDA score (above 4) and cladogram (data not shown). Muribaculaceae and Undentified Ruminococcaceae were the two that have been specifically modulated among such huge population, and accordingly, a prebiotic and restorative effect against CY by CPPF were performed (Figure 7). Briefly, the bacteria of order *Eubacteriales* were mainly regulated by CPPF, including genus *Oscillibacter, Ruminococcus, Papillibacter* in family Oscillospiraceae, and Lachnospiraceae.

#### The Determination of SCFAs

**FIGURE 8:**
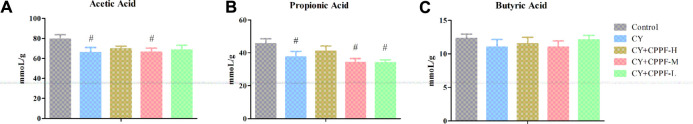
The effect of CPPF on the levels of SCFAs including **(A)** acetic acid, **(B)** propionic acid and **(C)** butyric acid in ileum tissue; C was the Control group, CP was the CY group, CP200 was the CY + CPPF-H group, CP100 was the CY + CPPF-M group, and CP50 was the CY + CPPF-L group; N = 7.

It has been revealed that inulin can be metabolized into SCFAs by microbial fermentation. The reduced gut lumen pH by SCFAs could inhibit the proliferation of pathogenic bacteria and stimulate intestinal peristalsis. These acids could be further utilized by intestinal epithelial cells for protecting the integrity of barrier function (Meijer et al., 2010). In this study, SCFAs concentrations in the cecal content, the position rich in bacterial community, were detected. The levels of acetic (Figure 8A) and propionic acids (Figure 8B) were decreased significantly by CY (*p* < 0.05), and the high dosage of CPPF could reverse this reduction, even without statistical difference. The butyric acid showed scarce change among different groups, but that in CPPF groups displayed an underlying increase (*p* > 0.05), as shown in Figure 8C.

## Discussion

As a common medicinal material, *C. pilosula* and its polysaccharide component have presented versatile pharmacological activities. Particularly, the polysaccharide from *C. pilosula* have been evidenced possessing both prebiotic and immunomodulatory activities *in vitro* or *in vivo*, by a range of previous studies (Peng et al., 2018; Deng et al., 2019) and experimental results in our group, including crude polysaccharide CPP (Fu et al., 2018a), inulin-type fructan CPPF (Fu et al., 2018b) and pectic polysaccharides (Zou et al., 2019). Considering its macromolecular and non-digestible properties in upper gastrointestinal tract, such natural polysaccharides can reach to intestine without degradation because of the limited hydrolysis enzymes in human (Wilson and Whelan, 2017; García Gamboa et al., 2018). Considering the modulation effects of *Codonopsis*-derived polysaccharides (Zhou et al., 2015; Li et al., 2018; Zou et al., 2019) or inulin (Vogt et al., 2015; Akram et al., 2019) on intestinal mucosal immune, the intestinal mucosa and/or gut microbiota were considered as a potential way that CPPF probably act.

Studies have demonstrated that CY destroys intestinal mucosal system by reducing barrier function, injuring intestinal villi and crypts, inhibiting secretion of sIgA and damaging Peyer’s patch (Miao et al., 2015; Zhao et al., 2016; Wang Z et al., 2019). In gastrointestinal mucosal barrier, goblet cells continually produce mucins to replenish and maintain the mucus barrier, where some chemical composition like sIgA is higher concentrated (Ouwerkerk et al., 2013; Cornick et al., 2015). The Muc2 and sIgA secretion were facilitated by CPPF, in accordance with other inulin reported before (Ito et al., 2008; Nagata et al., 2018). It could be directly resulted from activation of mucosal receptors cooperated with downstream signal pathways, or gut-associated lymphoid tissues (GALTs). Secretion cells, such as goblet cell, Paneth cells and enteroendocrine cells could also be involved (Kim and Ho, 2010; Vogt et al., 2015). It also possibly came from the intestinal bacteria change in an indirect way (Kim and Ho, 2010), that the abundance of certain mucus-degrading bacteria could be reduced (Schroeder et al., 2018; van den Abbeele et al., 2018). Thus, detailed composition changes in gut microbiota were analyzed.

Tissues with high proliferation rate, like intestinal epithelia cells, are vulnerable to CY because of its ability of killing dividing cells. Those damaged cells and produced reactive oxygen species (ROS) will induce apoptosis and up-regulate inflammatory cytokines (Taminiau et al., 1980; Moore, 1984; Sonis, 2004). There are numerous studies indicated that the treatment of CY could promote inflammatory cytokine secretion like IL-1β, TNF-α of porcine epithelial cells (Lee and Kang, 2017) or in small intestine (Shi et al., 2017; Wang X et al., 2019). Moreover, commercial inulin from Mexican blue agave (*Agave tequilana* Weber var. azul) or Chicory have been used as nutritional supplements with an anti-inflammatory effect in intestine (Rivera-Huerta et al., 2017; Akram et al., 2019; Zhao et al., 2019). However, it was very likely the first time showing the active target location of inulin from natural medicinal plants. Some studies indeed found an anti-inflammatory effect in intestinal mucosa of polysaccharide from *C. pilosula* (Zhou et al., 2015; Chen, 2016; Li et al., 2018). But no comprehensive investigation was declared that which intestinal part it can work on. Combing with previous studies of LCP-II-I (Huang C et al., 2017), we now have sufficient experimental evidence proving that these polymers could be active only before colon *in vivo*, since they could already been degraded or fermented in colon. In addition, the main factor of fermentation rate of inulin could be the DP variation (Gibson and Delzenne, 2008). Zhao et al. (2019) revealed that 28 days of short chain fructooligosaccahrides (scFOS) with maximum DP of 6, could down-regulate IL-1β expression in jejunal mucosa, but not active in ileum. Comparatively, the DP of CPPF in our study was higher (about 2–17) (Fu et al., 2018b), and could be degraded much slowly and even far in intestine tract. That was probably the main reason that CPPF displayed well on anti-inflammatory effect in both jejunum and ileum.

Polysaccharides from marine animals, fruits, vegetables and natural medical plants, have been found with high fermentable and modulatory effect by gut microbiota (Bosscher et al., 2006; Xu et al., 2013; Shoaib et al., 2016; Shang Q et al., 2018). Although immunosuppressed mice were treated with CPPF for only 7 days, there was still bacterial community change observed in our research. Some similar studies (Chen et al., 2017; Moens and De Vuyst, 2017; Zhu et al., 2017; Hoving et al., 2018; Baxter et al., 2019; Li et al., 2019) also manifested that the abundance of *Clostridium*, *Deferribacteres*, Ruminococcaceae, Lachnospiraceae or *Oscillospira* were regulated by several weeks of inulin treatment. As one of probiotics, *Ruminococcceae* has been well reported with a responsible relation to polysaccharides degradation, like fucoidans and inulin (Shang et al., 2016; Moens and De Vuyst, 2017). In this study, a remarkable increase of *unidentified* Ruminococcaceae was demonstrated by CPPF, which proved it with a prebiotic activity. CPPF also displayed an ameliorated effect by decreasing the amount of *Mucispirillum schaedleri*, which is parasitic in intestinal mucous layer, and can easily translocate in intestinal mucosa and activate NF-kB or PPAR-delta receptors to trigger inflammatory response (Loy et al., 2017). The *Oscillospira* recovered by CPPF is one of the butyrate-producing bacteria, and are usually considered as protector against several pathogens by acidifying intestinal environment. This recovery has also been achieved by polysaccharides from purple sweet potato on immunosuppressed mice (Tang et al., 2018). Moreover, it was found that the *Blautia* in the family of Lachnospiraceae was promoted after CPPF administration as shown in Figure 5D. It has been proved that the relative abundance of *Lachnospiraceae* has significant correlation with host physiological dysfunctions, such as obesity, diabetes, cancer, and various inflammatory diseases, and could be an underlying probiotic (Liu et al., 2021).

Furthermore, the relative abundance of *Lactobacillus* was displayed an opposite trend with our previous results, that it was reduced relatively in mice treated with inulin, which was also showed in study of Hoving et al. (2018). After reviewing the overall microflora data, we inferred that it might be due to the different *Lactobacillus* species that we used in previous and current study. And the overwhelming reduction by CY on the abundance of other bacteria could lead to a relatively increase of *Lactobacillus*. A comparatively lower abundance in CPPF groups may therefore be observed. The dose could be another factor leading to a lower prebiotic effect. No significant change on the relative abundance of Bifidobacteriaceae and Lactobacillus was observed after even above 1.25 g/kg of inulin/FOS administration (Zhu et al., 2017).

As one of the non-digested polymers, inulin can reach to intestine intactly, and serve as energy source for beneficial bacteria that generate metabolites like SCFAs. The increase of butyric acid, the main one utilized by colonocytes, was related to a higher abundance of genera Ruminococcaceae (Biddle et al., 2013; Fernández et al., 2016), which produces butyrate as most abundant family in the order *Clostridiales*. Additionally, Ruminococcaceae was indicated with positive correlation with butyrate/SCFA ratio *in vivo* (Biddle et al., 2013). And the *Oscillibacter*, *Ruminococcus* and *Papillibacter,* belonging to family Ruminococcaceae, were all promoted by CPPF in our study. This alteration on bacterial composition could be the main reason of the certain high butyric acid in CPPF group compared with CY group, similar with previous studies (Moens and De Vuyst, 2017; Baxter et al., 2019). The acetic acid is produced by most enteric bacteria. Its dramatically drop in cecal content could because of the lower bacterial OTUs induced by CY. While the less restoration in CPPF groups could be due to the lower abundance of acetic acid-producing bacteria, like *Lactobacillus* (Fernández et al., 2016) (Figure 5). The relative abundance of genera *Bacteroides*, who converts sugars to beneficial metabolites, like propionic acids (Biddle et al., 2013; Fernández et al., 2016), were higher in CPPF-H group than CY group, and was coincident with results of SCFAs levels. Propionic acid could be produced mainly by ketone fermentation (e.g., fructose, arabinose and tagatose) *in vitro* (Hu et al., 2013). The abundant fructose in CPPF could be one of the reasons of its higher concentration. Comparatively, the acetic and butyric acids are produced mainly by aldehydes fermentation (e.g., glucose, galactose, mannose and xylose) (Hu et al., 2013). These changes on acetic and butyric acids are likely coming from bacteria modulated by CPPF in current research, rather than its degradation metabolites, as CPPF is consisted of a lower amount of glucose relatively to fructose. However, such advantageous SCFAs were not promoted by CPPF. The possible reason is that the mice were not intervened enough to show a significantly change on gut flora and carbohydrate fermentation. And the relative abundance of those SCFAs-producing bacteria could be influenced by complicated micro-environment, which is also a challenge of studies on microbiota and polysaccharide degradation.

## Conclusion

After identification of the structural and prebiotic features, the inulin-type fructan from *Codonopsis pilosula* (CPPF) was further studied on its modulatory effect on intestinal mucosa and gut microbiota. It was demonstrated that the intestinal mucosal immune of CY-induced immunosuppressed mice was restored by CPPF through promotion of Muc2 and sIgA secretions. A new view that the intestinal anti-inflammatory effects of CPPF only worked before colon was also provided. The modulatory effect on gut microbiota was also investigated, that CPPF increased the relative abundance of *Oscillibacter*, *unidentified Ruminococcus*, Lachnospiraceae and decreased that of *Deferribacteres*. It was suggested that CPPF could be considered as the main bioactive component of *C. pilosula* polysaccharide, being used as a potential prebiotic with improving intestinal mucosal immune, anti-inflammatory and microbiota modulation activities.

## Data Availability

The original contributions presented in the study are included in the article/Supplementary Material, further inquiries can be directed to the corresponding authors.
