# Supplementary material for: Restorative Effects of Inulin From Codonopsis pilosula on Intestinal Mucosal Immunity, Anti-Inflammatory Activity and Gut Microbiota of Immunosuppressed Mice
Source: Front Pharmacol. 2022 Feb 14;13:786141. doi: 10.3389/fphar.2022.786141 (PMC8882912; doi:10.3389/fphar.2022.786141)
Supplement: Supplementary file 1 [file DataSheet1.pdf]

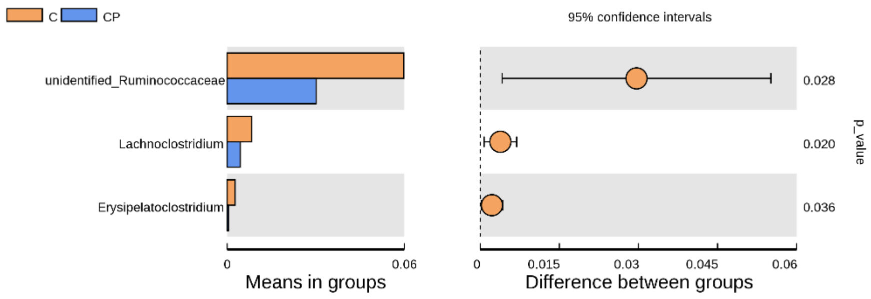

Figure S1. T-test results of species differences on genus level between groups. C was the Control group, CP was the CY group; N=7.

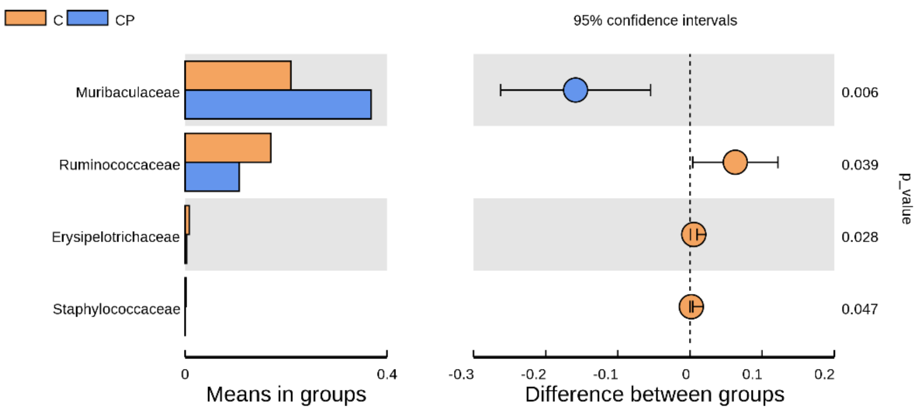

Figure S2. T-test results of species differences on family level between groups. C was the Control group, CP was the CY group; N=7.

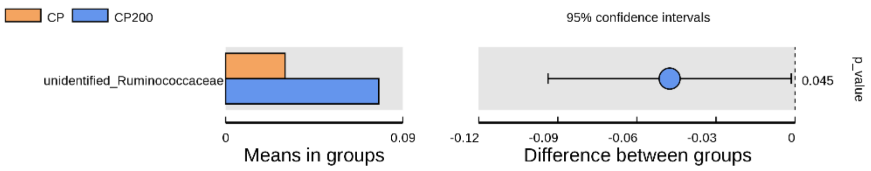

Figure S3. T-test results of species differences on genus level among groups. CP was the CY group, CP200 was the CY+CPPF-H group; N=7.

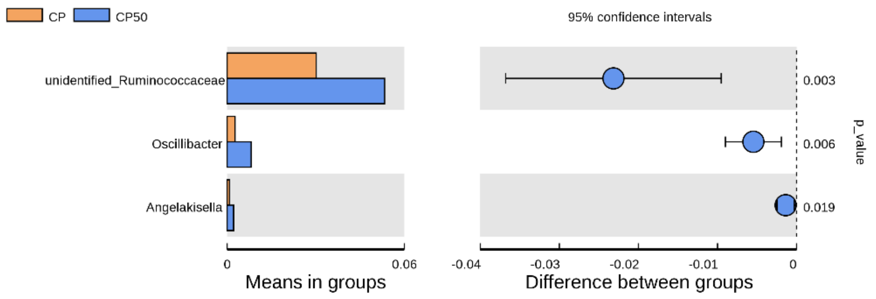

Figure S4. T-test results of species differences on genus level between groups. CP was the CY group, CP50 was the CY+CPPF-L group; N=7.

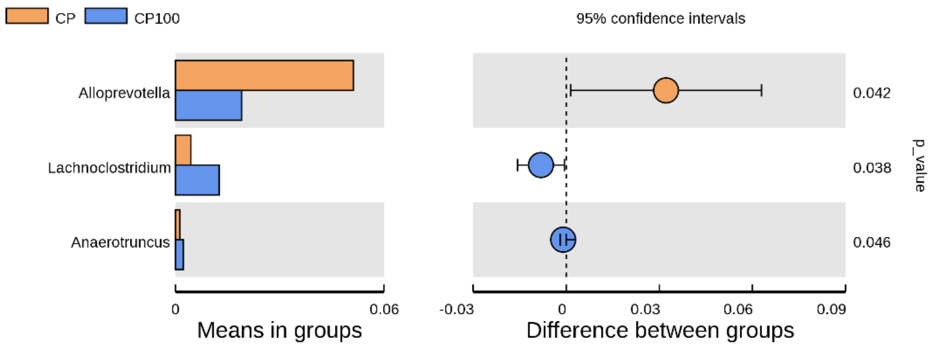

Figure S5. T-test results of species differences on genus level between groups. CP was the CY group, CP100 was the CY+CPPF-M group; N=7.
